# Supplementary figures and images for: Glucocerebrosidase deficiency leads to neuropathology via cellular immune activation
Source: PLoS Genet. 2024 Nov 11;20(11):e1011105. doi: 10.1371/journal.pgen.1011105 (PMC11581407; doi:10.1371/journal.pgen.1011105)

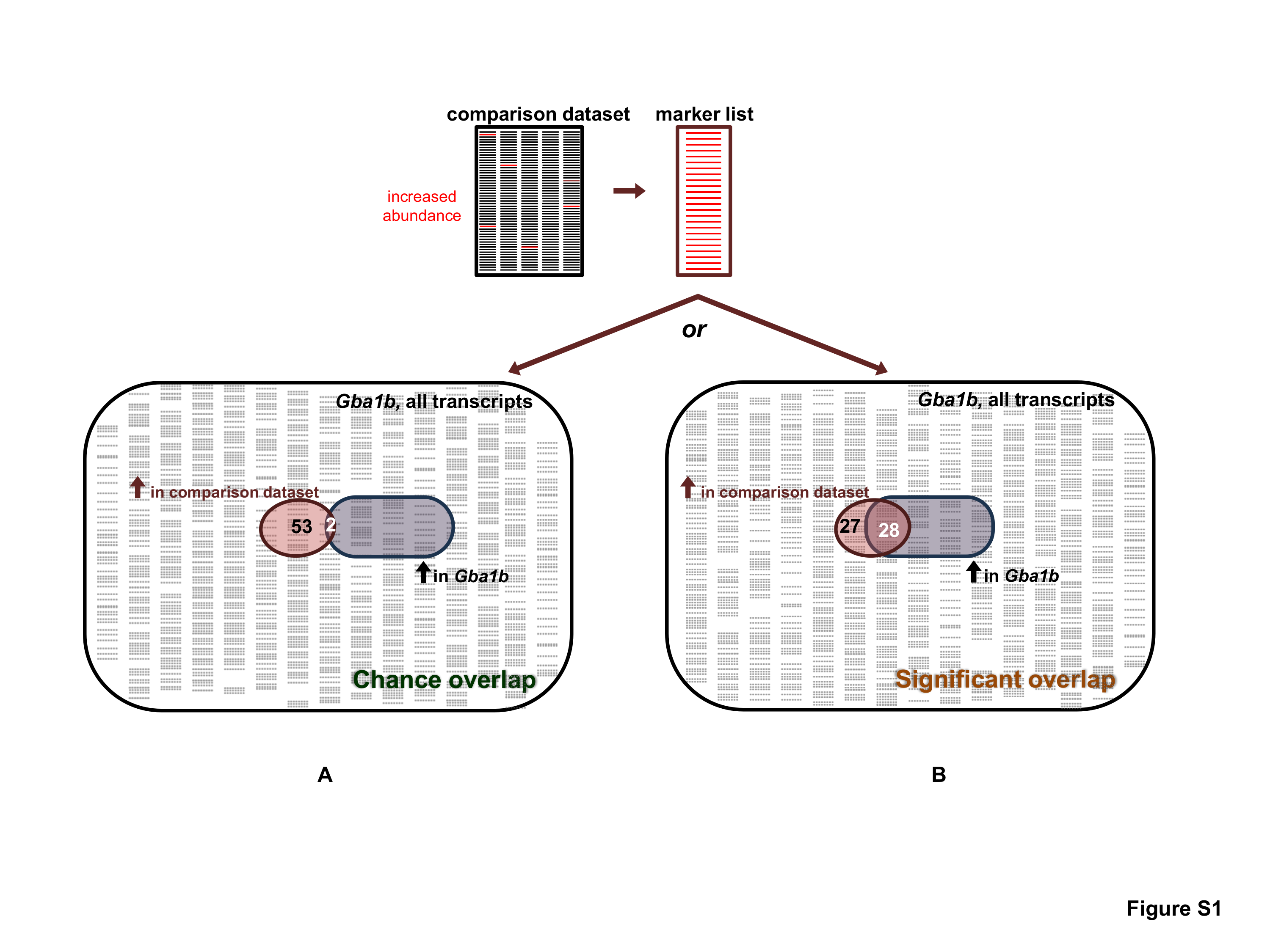

Supplement: S1 Fig — The diagram illustrates our method of comparing Gba1b mutant RNA-Seq and proteomic data to available datasets on immune response and immune cell markers, showing two scenarios. See Materials and Methods for full details. The example is based on the marker list from the immune meta-analysis study [28], which includes 55 transcripts. (A) Chance-level overlap (no enrichment). Two transcripts appear both in the marker list and in the list of transcripts increased in abundance in Gba1b mutants. This is consistent with chance-level overlap, as 2/55 transcripts approximates the whole-dataset percentage of 3.5% transcripts with increased abundance. (B) Significant overlap. Twenty-eight transcripts appear both in the marker list and in the list of transcripts increased in abundance in Gba1b mutants. This is a statistically significant overlap, as 28/55 transcripts is 50.9%, more than 14 times the chance-level overlap of 3.5%. (TIF) [file pgen.1011105.s001.tif]

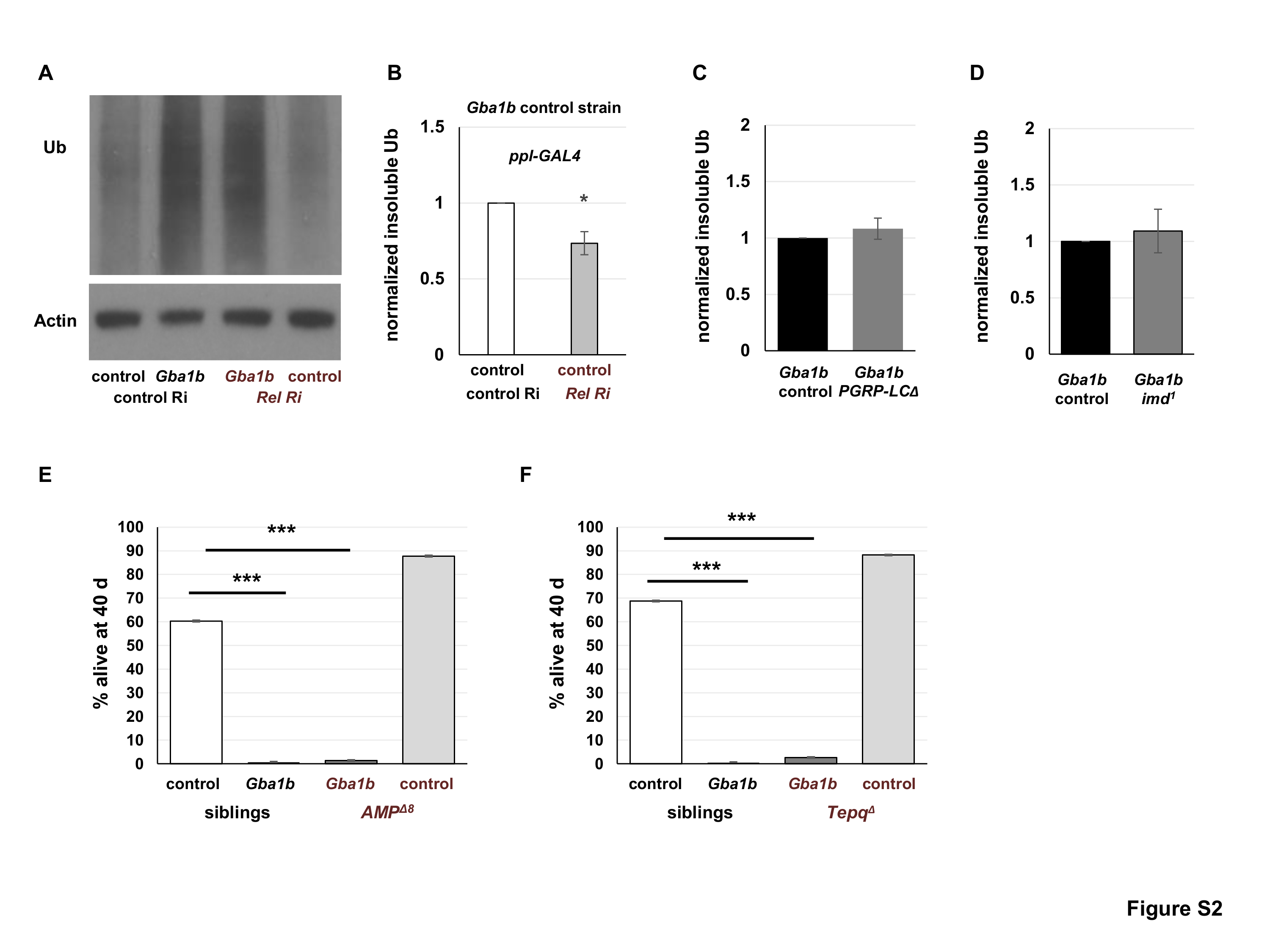

Supplement: S2 Fig — (A) Immunoblot of insoluble ubiquitinated proteins from heads of Gba1b mutants with and without Relish RNAi driven by ppl-GAL4. (B) Quantification of panel A for Gba1b revertant controls with vs. without Relish RNAi. The same comparison for Gba1b mutants is shown in Fig 2B. *p < 0.05 by Student’s t test. (C) Quantification of insoluble ubiquitinated proteins in heads from Gba1b mutants with and without loss of PGRP-LC function. (D) Quantification of insoluble ubiquitinated proteins from Gba1b mutants (whole flies) with and without the hypomorphic imd1 mutation. (D) Percentage of flies alive at day 40, Gba1b mutants and controls with or without AMPΔ8 mutation. n = 7 vials per group, 21 flies per vial, total of at least 145 flies per genotype. (E) Percentage of flies alive at day 40 Gba1b mutants and controls with or without TepqΔ mutation. n = 8–10 vials per group, 18–21 flies per vial, total of 170–200 flies per genotype. *** p < 0.005 by one-way ANOVA with Dunnett’s T3 multiple comparisons test. (TIF) [file pgen.1011105.s002.tif]

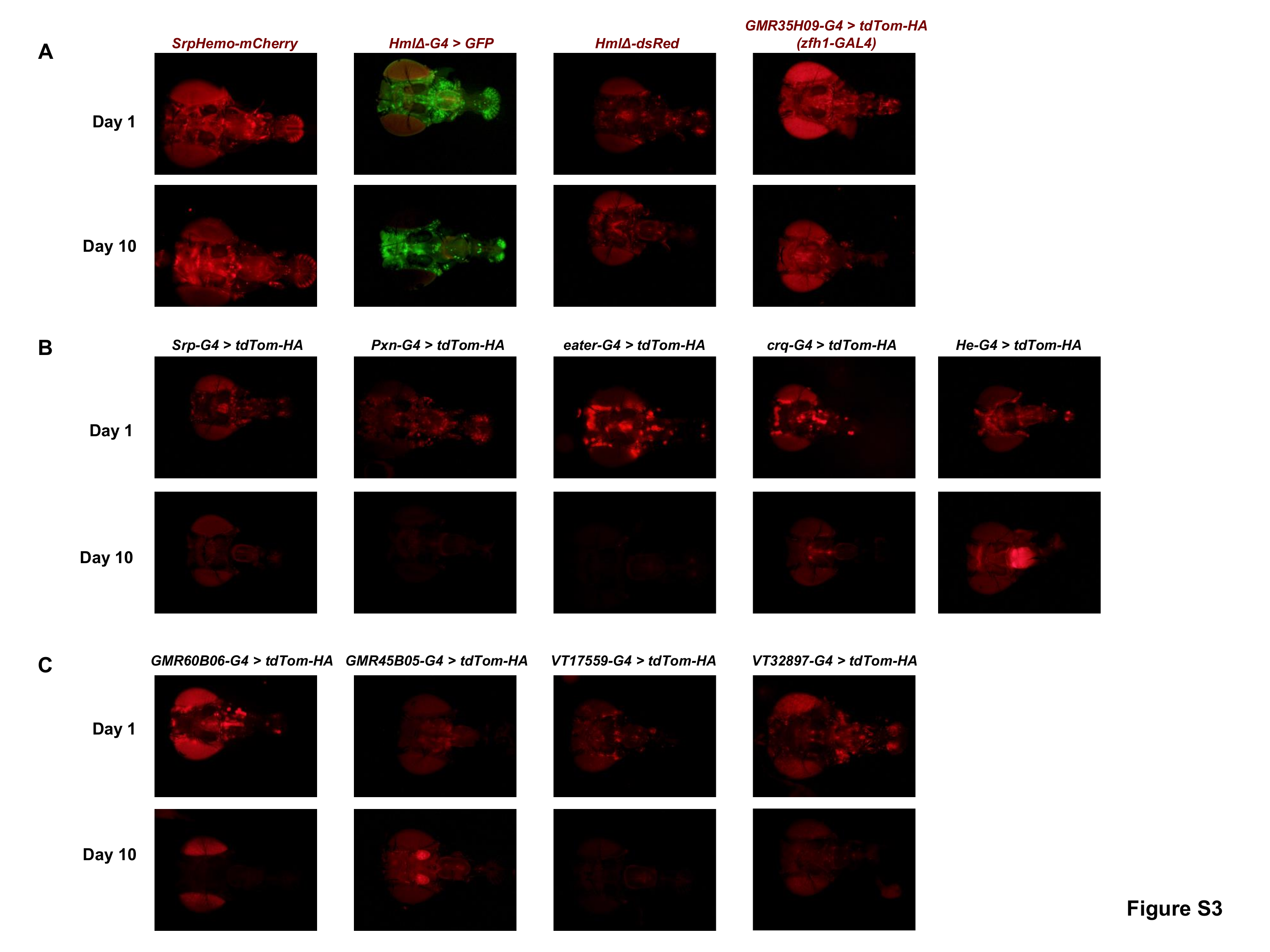

Supplement: S3 Fig — Heads were matched for sex and imaged at 1 or 10 d of age, then evaluated for fluorescent signal in a distribution consistent with macrophages. Heads were imaged caudal side down, with the ventral aspect to the left. (A) Markers and GAL4 drivers that maintain macrophage expression through 10 d of adult life. (B) Well-known macrophage GAL4 drivers that lack visible macrophage expression at 10 d of age. (C) Other macrophage GAL4 drivers with expression that lack visible macrophage expression at 10 d of age. (TIF) [file pgen.1011105.s003.tif]

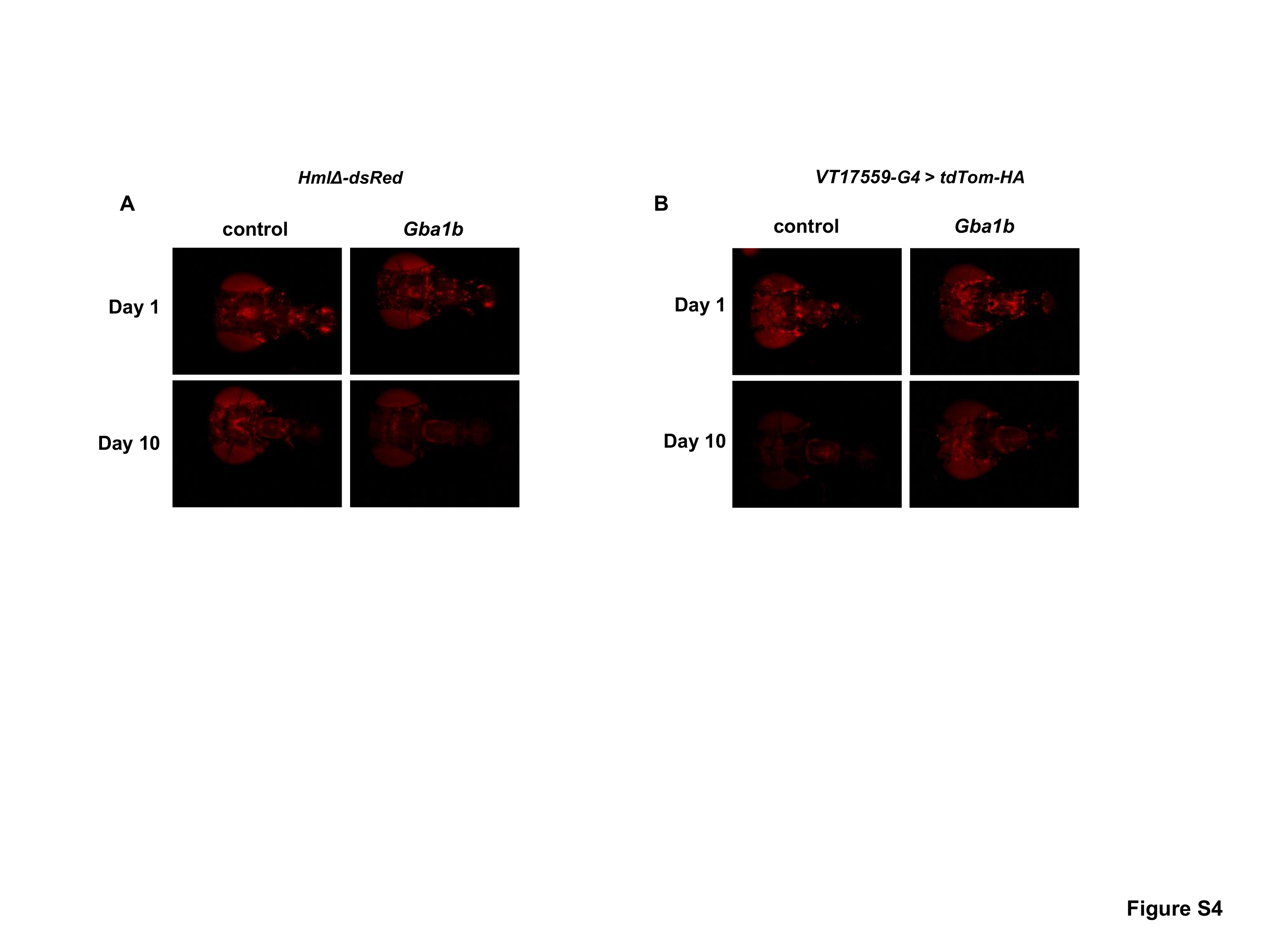

Supplement: S4 Fig — (A) The HmlΔ promoter directly driving dsRed expression in control flies and Gba1b mutants gives the same result as HmlΔ-GAL4. (B) VT17559-GAL4 contains promoter sequence from the Lis-1 gene, an additional marker of macrophage activation. Like zfh1-GAL4, this marker is elevated in Gba1b mutants vs. controls at 10 d of age. (TIF) [file pgen.1011105.s004.tif]

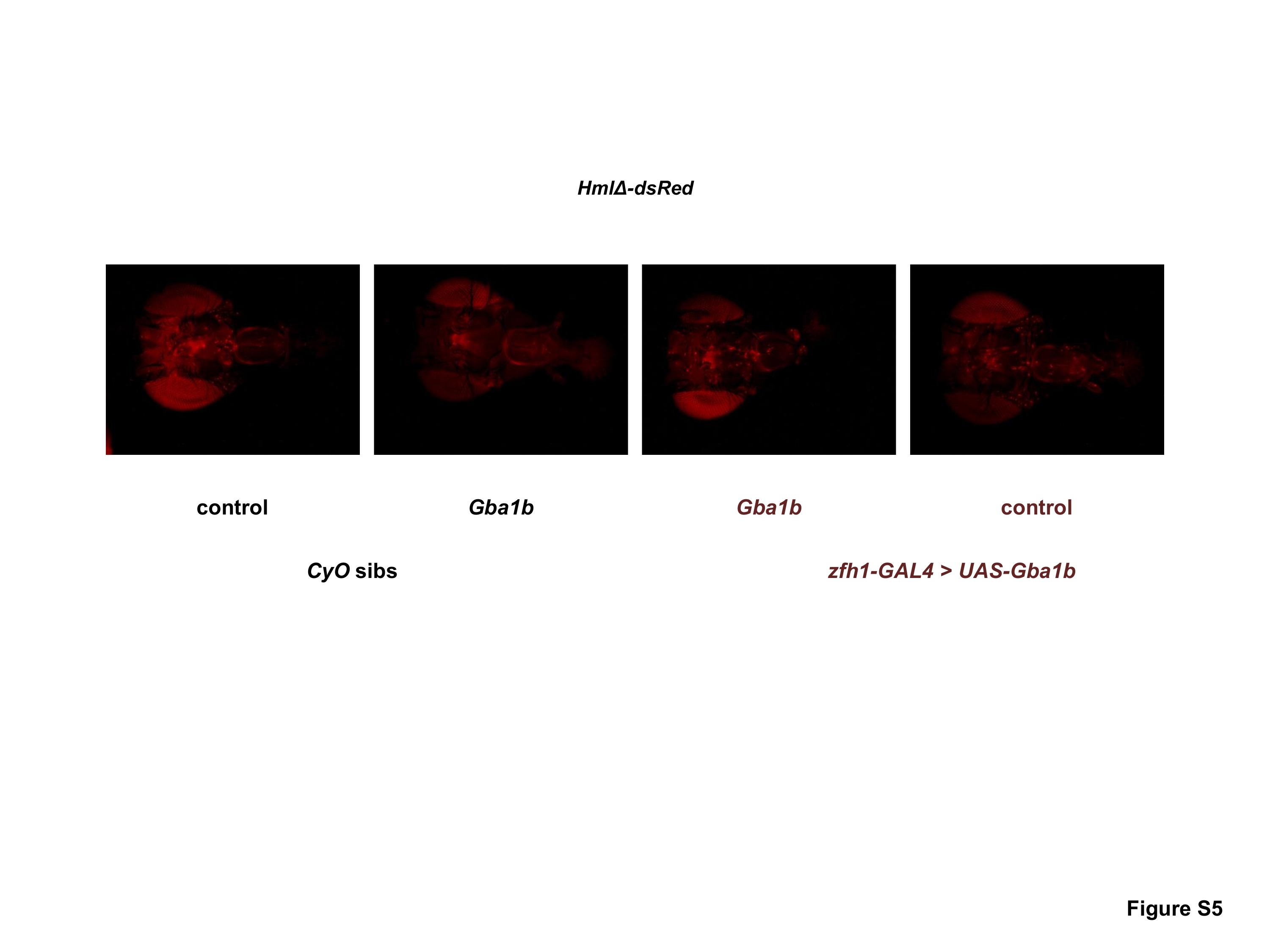

Supplement: S5 Fig — The experiment was performed similarly to the rescue experiment shown in Fig 6A with zfh1-GAL4 driving UAS-Gba1b. In this case, the HmlΔ-dsRed marker was substituted for zfh1-lexA > tdTom. Flies were 10 days old. (TIF) [file pgen.1011105.s005.tif]

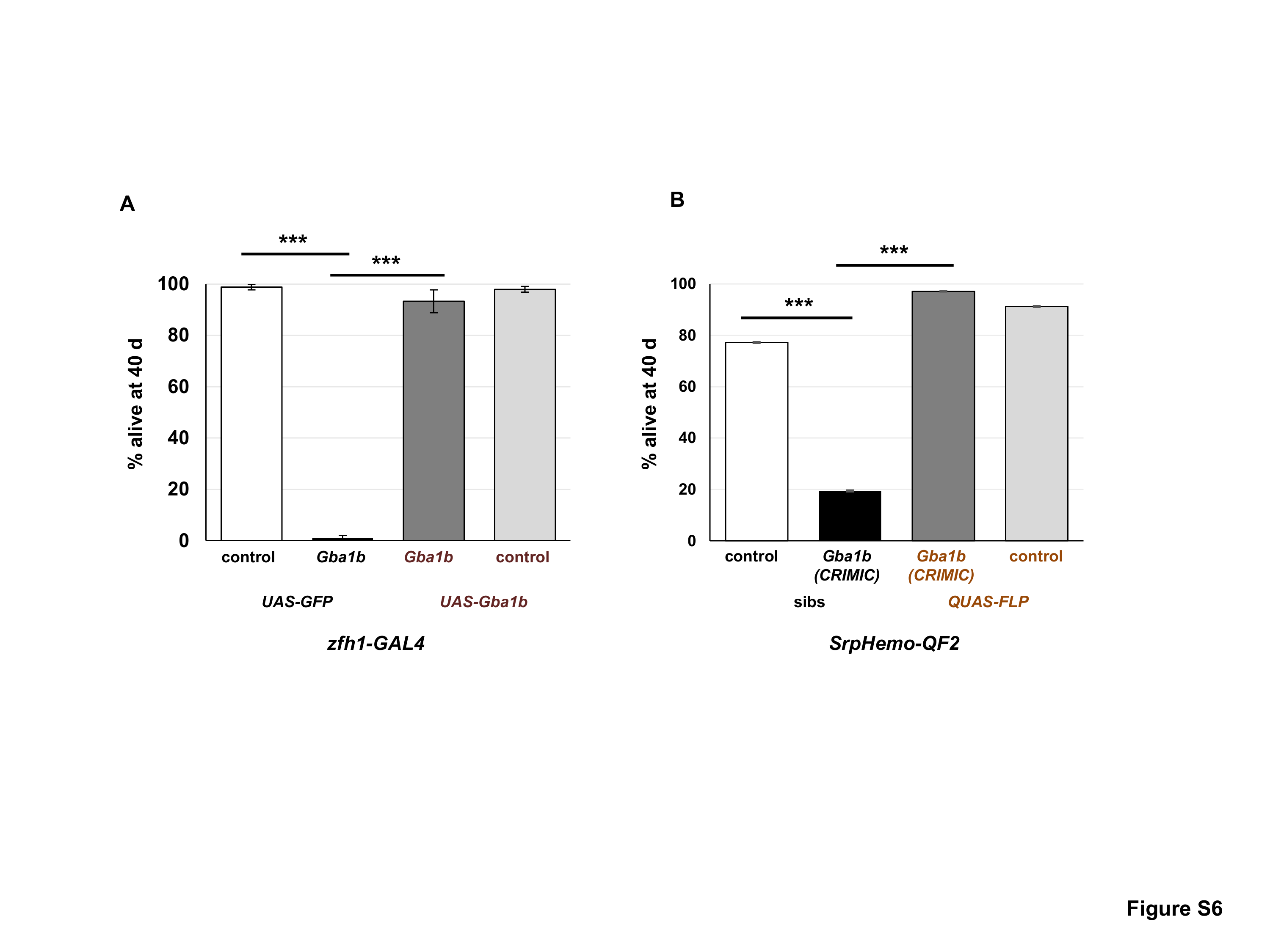

Supplement: S6 Fig — (A) Percentage of flies alive at day 40 in Gba1b mutants and controls with or without rescue expression of Gba1b under control of zfh1-GAL4. n = 4–6 vials per group, 10–24 flies per vial at 20 d, total 150–200 flies per genotype. (B) Percentage of flies alive at day 40 in Gba1b mutants and controls with or without restored expression of Gba1b in macrophages under control of SrpHemo-QF2. n = 6–7 vials per group, 10–24 flies per vial at 20 d, total 120–136 flies per genotype. ***p < 0.005 by one-way ANOVA with Dunnett’s T3 multiple comparisons test. (TIF) [file pgen.1011105.s006.tif]

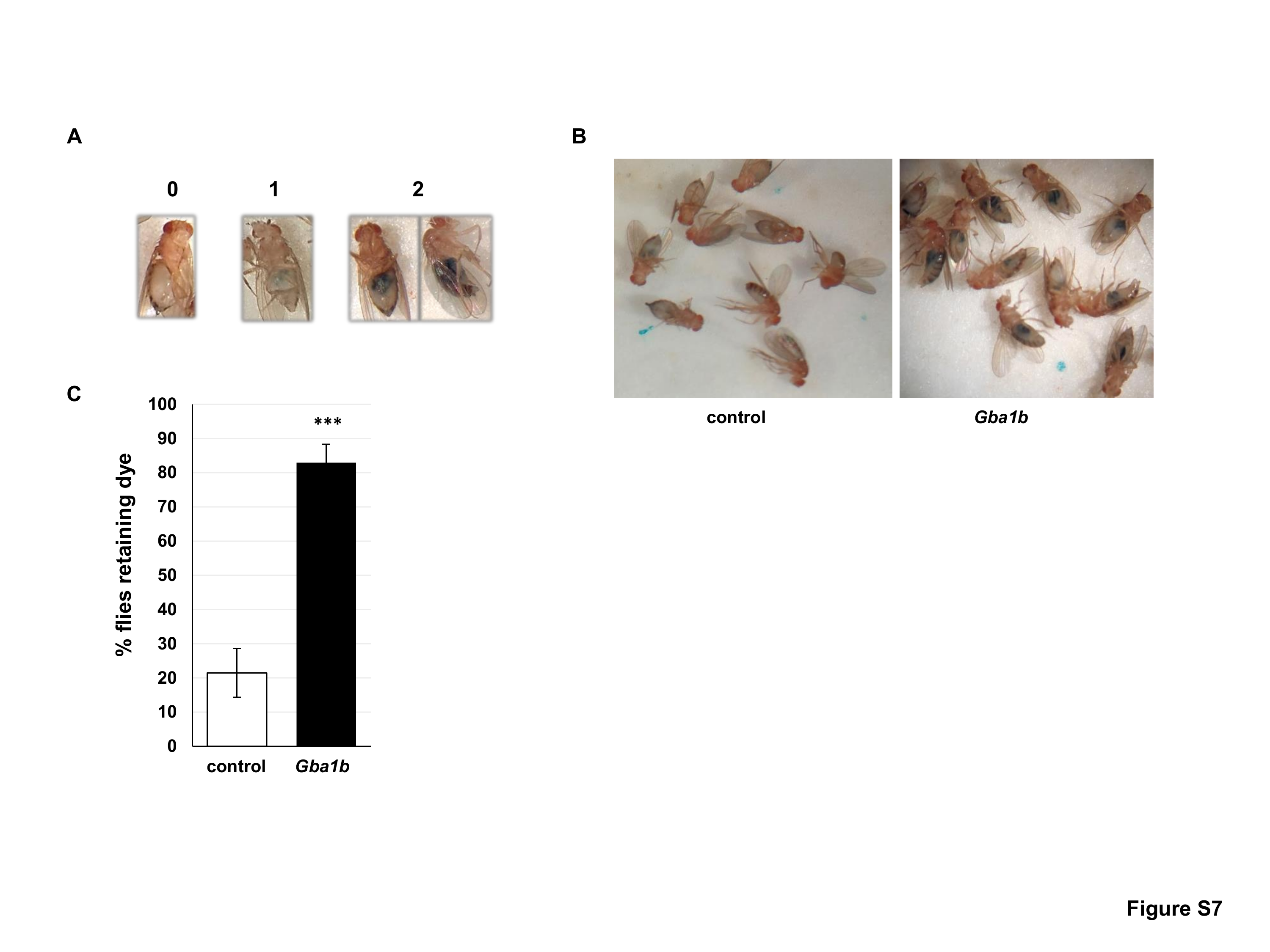

Supplement: S7 Fig — (A) Photos of gut transit assay scoring categories. Flies with a score of 2 (blue dye strongly visible in abdomen) were considered to have impaired gut transit, while flies with a score of 1 (faint to moderate blue) or 0 (no blue) are considered to have unimpaired gut transit. (B) Examples of control and Gba1b flies 2.5 h after switching from dyed to regular food. (C) Percent of flies retaining dye after 2.5 h. The Gba1b mutants and control flies used in panel C also bear mCherry RNAi. ***p < 0.005 by Student t test. (TIF) [file pgen.1011105.s007.tif]

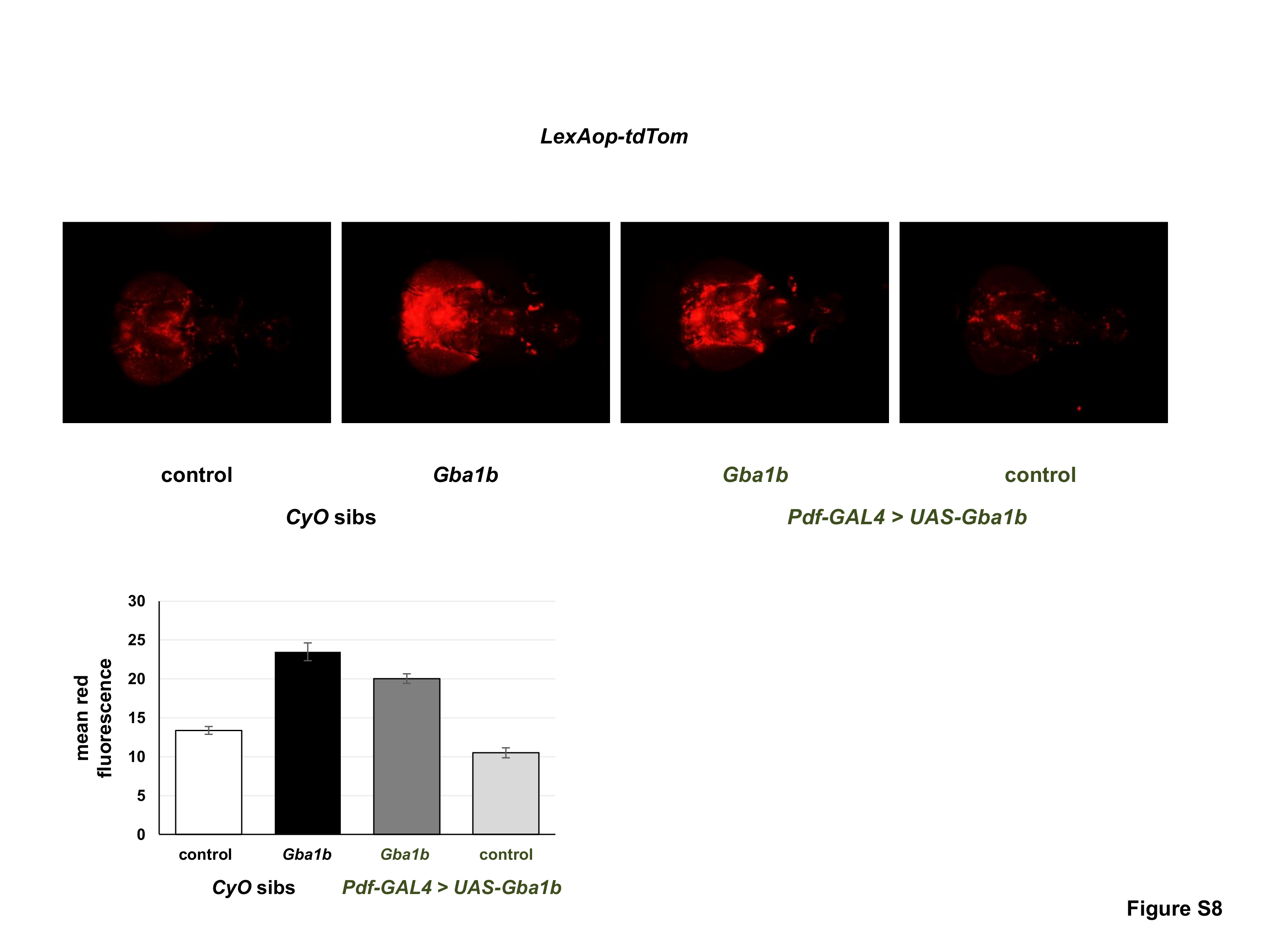

Supplement: S8 Fig — Macrophage activation was visualized using zfh1-lexA driving lexAop-tdTom-HA (n = 6–8 heads per genotype). Flies were 10 d old. Graph is the mean fluorescence from male flies. Significance was tested using one-way ANOVA with Dunnett’s T3 multiple comparisons test and no significant difference was found between Gba1b mutants with or without Pdf-GAL4 driven Gba1b expression. (TIF) [file pgen.1011105.s008.tif]

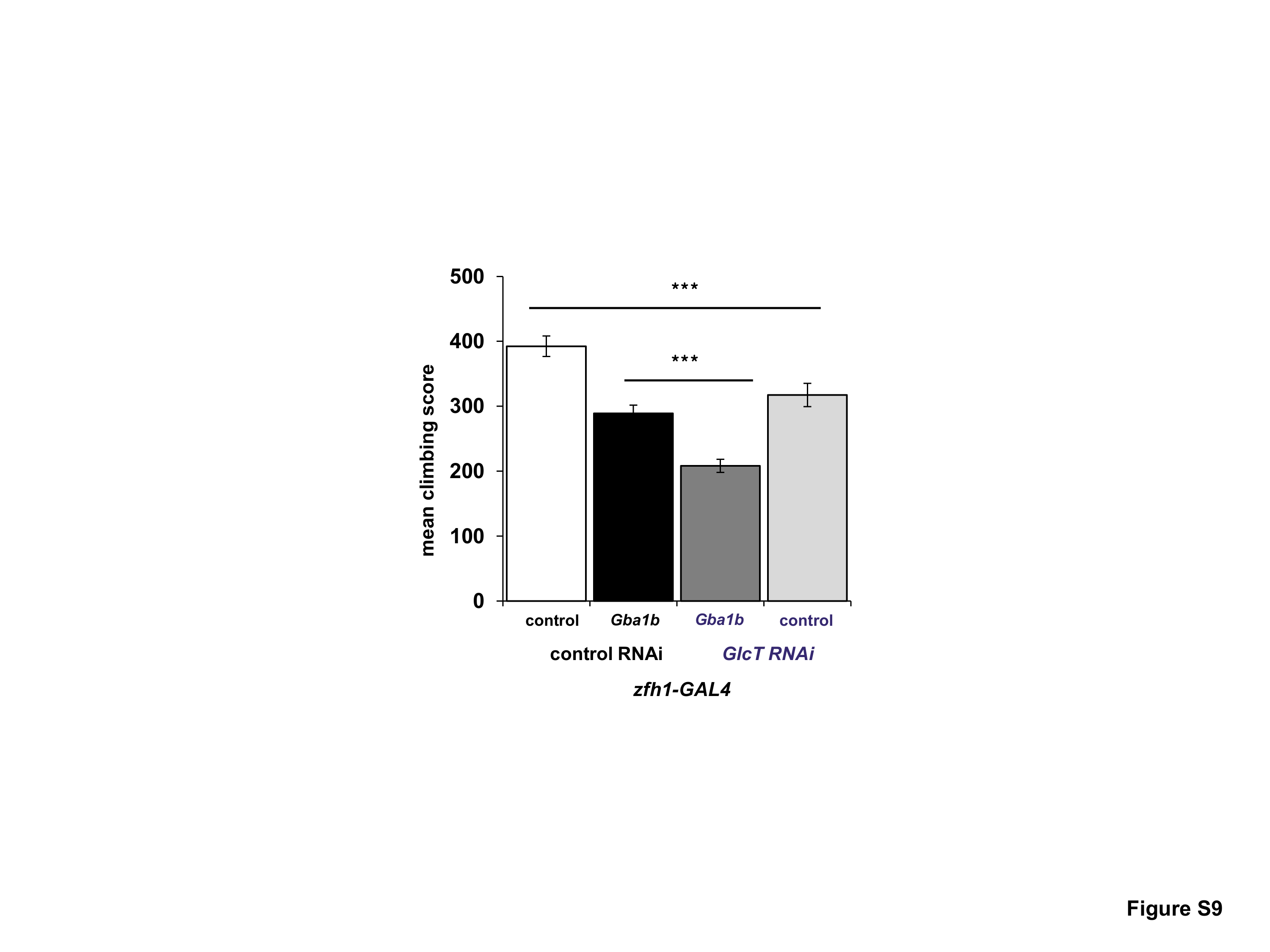

Supplement: S9 Fig — Climbing was measured using the RING assay in male and female flies at 15–17 d of age. ***p < 0.005 by one-way ANOVA. (TIF) [file pgen.1011105.s009.tif]
